# Alpine speciation and morphological innovations: revelations from a species-rich genus in the northern hemisphere

**DOI:** 10.1093/aobpla/plab018

**Published:** 2021-04-15

**Authors:** Yazhou Zhang, Jianguo Chen, Hang Sun

**Affiliations:** 1 CAS Key Laboratory for Plant Biodiversity and Biogeography of East Asia, Kunming Institute of Botany, Chinese Academy of Sciences, No. 132, Lanhei Road, Kunming 650201, Yunnan, China; 2 University of Chinese Academy of Sciences, Beijing 100049, China

**Keywords:** Adaptive evolution, alpine speciation, diversity, endemism, morphological innovations, *Saussurea*

## Abstract

A large number of studies have attempted to determine the mechanisms driving plant diversity and distribution on a global scale, but the diverse and endemic alpine herbs found in harsh environments, showing adaptive evolution, require more studies. Here, we selected 466 species from the genus *Saussurea*, one of the northern hemisphere’s highest-altitude plant genera with high species richness and striking morphological traits, to explore the mechanisms driving speciation and adaptative evolution. We conducted phylogenetic signals analysis and ancestral character estimation to explore the phylogenetic significance of ecological factors. Moreover, we used spatial simultaneous autoregressive (SAR) error models, modified *t*-tests and partial regression models to quantify the relative effects of ecological factors and morphological diversity upon diversity and endemism of *Saussurea*. Phylogenetic analyses reveal that geological influences and climate stability exhibit significant phylogenetic signals and that *Saussurea* originated at a relatively high elevation. Regression models indicate that geological influences and climatic stability significantly affect the diversity and endemism patterns of *Saussurea* and its morphological innovations. Moreover, morphological innovations in an area show significant contributions to the local diversity and endemism of *Saussurea*. We conclude that geological influences (mean altitude and topographic heterogeneity), glacial–interglacial climate stability and phylogenetic conservatism have together promoted the speciation and adaptive evolution of the genus *Saussurea*. In addition, adaptively morphological innovations of alpine species also promote diversification in local regions. Our findings improve the understanding of the distribution pattern of diversity/endemism and adaptive evolution of alpine specie in the whole northern hemisphere.

## Teaser

Focusing on the diversity, endemism and adaptation of alpine herbs, we study *Saussurea* species, one of the northern hemisphere’s highest-altitude plant genera. This study reveals the important effects of geological, glacial and phylogenetic factors on the diversity patterns of alpine taxa, which offers an excellent example to study and understand the diversity pattern, endemism pattern and adaptive evolution of alpine species in the northern hemisphere. This study also provides a meaningful attempt to explore spatial bias and phylogenetic uncertainties in a species-rich genus.

## Introduction

Alpine settings are an important habitat, partly due to their extremely rich biodiversity (Antonelli *et al.* 2018). When attempting to explain why there are so many species in alpine habitats, biologists have always focused on geological and climatic factors (Hoorn *et al.* 2013). Recent orogeny generated various biodiversity hotspots on a global scale, including the Qinghai–Tibetan Plateau (QTP; Favre *et al.* 2015; Xing and Ree 2017; Yu *et al.* 2019), the Andes (Hughes 2016; Esquerré *et al.* 2019) and south-east Asia (Merckx *et al.* 2015). Moreover, ice ages during the Pleistocene also had a dramatic impact on global biodiversity patterns. For example, in many mid- to high-latitude areas (e.g. North America and northern Europe), glaciations destroyed the local biodiversity (Webb and Bartlein 1992; Provan and Bennett 2008). In contrast, glaciations were great drivers of biodiversity in linear mountain ranges of temperate regions, such as the Andes (Sérsic *et al.* 2011; Hazzi *et al.* 2018), the Pyrénées (Liberal *et al.* 2014), the Southern Himalayas (Fan *et al.* 2013; Luo *et al.* 2016) and the Southern Alps (Weston and Robertson 2015). Due to their particular geological and climatic characteristics, such as diverse topography, heterogeneous climatic types and long-term climatic stability (Hoorn *et al.* 2013), mountains host exceptional plant biodiversity (Körner 2003). Although great efforts have been put into exploring the mechanisms driving plant diversity and distribution (e.g. Wen *et al.* 2014; Wang *et al.* 2017; Shrestha *et al.* 2018; Xu *et al.* 2019a) on a global scale, the diversity and endemism of alpine herbs require additional studies. Further, there is particular value in examining drivers of mountain biodiversity at multiple levels, such as the ecological level and the phylogenetic level.

Plants in mountain regions are exposed to extreme environmental stresses, including low temperature, poor soil quality, strong wind and UV radiation (Körner 2003). However, over their long evolutionary history, mountain plants have developed particular adaptive strategies, including highly specialized phenological, morphological and physiological mechanisms and structures (Körner 2003; Nagy and Grabherr 2009; Sun *et al.* 2014). For example, in terms of physiological strategies, some plants can effectively accumulate flavonoids in particular organs (e.g. leaves, bracts, fruits) in order to resist strong UV radiation (e.g. Omori and Ohba 1996; Omori *et al.* 2000). While, in terms of morphological strategies, there have been key evolutionary innovations in many alpine plants which could be a result of convergent evolution. For example, the ‘greenhouse’ morphology, which is defined as the presence of large translucent or coloured bracts that cover the inflorescences (Ohba 1988) and which can increase temperature within inflorescences and protect reproductive organs from rain and UV radiation (e.g. Song *et al.* 2013), has been recorded in >10 plant families (Yang and Sun 2006; Xu *et al.* 2014). The cushion morphology, which can modify the micro-environment, thus moderating severe alpine environmental conditions, has been found in >1300 species belonging to 63 families (Aubert *et al.* 2014). Other commonly found morphological traits in alpine plants include the so-called ‘woolly plants’, ‘nodding flower plants’, ‘airbag plants’ and ‘moving plants’ (Sun *et al.* 2014). All these specialized traits are key morphological innovations for alpine plants in their long evolutionary history, and most of the adaptive mechanisms of these traits have been thoroughly examined (Sun *et al.* 2014 and references therein). Notably, morphological innovations have been verified as a key process driving species diversification (Maurin *et al.* 2014; Fernandez-Mazuecos *et al.* 2019). However, few studies have examined the link between the geographical distributions of these morphological innovations and large-scale ecological characters and diversity (but see Boucher *et al.* 2016), even though such work could be valuable for understanding the speciation and diversification of alpine plants.

There are many well-known alpine biodiversity hotspots in the world, including the Himalayas, Andes and East Africa (Hoorn *et al.* 2013). Unlike the fragmented tectonic plates of the southern hemisphere, the continents of the northern hemisphere are relatively intact, resulting in different mountain system continuities in the two hemispheres (Billings 1974). In the north, the mountain floras are more closely linked, a fact supported by many biogeographic studies concerning long-distance migration, land bridges and so on, and this is also reflected in the large number of shared plant taxa in the arctic and alpine areas (Wen *et al.* 2014; Chen *et al.* 2018). The QTP, a key hotspot in the northern hemisphere, which contains the Himalayas (West-East), the Hengduan Mountains (HDM, South-North) and the Plateau proper, is the highest and largest plateau in the world and harbours one of the world’s richest temperate floras, with >12 000 species of vascular plants (Sun *et al.* 2014; Wen *et al.* 2014; Zhang *et al.* 2016). Known as the third pole of the world, the QTP is home to a large number of alpine taxa due to its vast range of microclimate types (Sun *et al.* 2014). The QTP is a ‘cradle’ of diversity, with the famous ‘out of Tibet’ hypothesis suggesting that many alpine taxa originated on the QTP and expanded to other regions (e.g. Deng *et al.* 2011; Li *et al.* 2014; Favre *et al.* 2016; Xu *et al.* 2019b). The QTP is also a ‘museum’, because many genera migrated onto the QTP and formed diversification centres or endemism centres in this region (Yue *et al.* 2009; Mao *et al.* 2010; Hou *et al.* 2016a, b). In addition, the ecological functions of the highly specialized morphological traits are now well understood thanks to the recent explosion of research on the QTP (Sun *et al.* 2014 and references therein). Exploring biodiversity based on the QTP is of great value in understanding the mechanisms underlying the distribution patterns and speciation of alpine taxa, especially in the northern hemisphere. In addition, linking morphological innovations to speciation could offer clues to the mechanisms of diversification since those innovations are driven by environmental conditions associated with geological and climatic factors, also driving species diversity (Nagy and Grabherr 2009; Sun *et al.* 2014; Wen *et al.* 2014).

*Saussurea* is one of the largest genera in the Asteraceae family, with ~460–490 herbaceous species widely distributing in the northern hemisphere (Raab-Straube 2017; Xu *et al.* 2019b). Species of this genus mainly occur in the alpine habitats of the Sino-Himalaya region and temperate regions of Asia (Chen 2015; Raab-Straube 2017). *Saussurea* is a typical alpine group and its uppermost altitudinal limit of ca. 6300 m is the highest location of seed plants on record (Raab-Straube 2017). According to phylogenetic analyses, *Saussurea* is a polyphyletic group with several parallel clades in the lineage, supporting island-like adaptive radiations in a continental setting and morphological convergences on the QTP (Wang *et al.* 2009; Wen *et al.* 2014). Furthermore, some species have been recently excluded from *Saussurea* with the aim of circumscribing a monophyletic genus based on the results of molecular phylogenies (Xu *et al.* 2019b). Moreover, *Saussurea* species have evolved a high diversity of specialized morphological traits adapting them to the different environmental stresses experienced in mountain regions. For example, many species from subgen. *Amphilaena* have greenhouse bracts (e.g. *S. velutina*, *S. obvallata*), many species in subgen. *Eriocoryne* are woolly (e.g. *S. medusa*, *S. leucoma*), while other species adopt cushion forms (e.g. *S. subulata*, *S. salwinensis*) or other specialized morphological traits (e.g. rosettes/stemless leaves). In addition, a recent study confirmed that *Saussurea* originated from the HDM during the early-middle Miocene and then migrated out of Tibet (Xu *et al.* 2019b). All of these characteristics make *Saussurea* an excellent model to study speciation, diversification and distribution of mountain species (also see Xu *et al.* 2019b).

In this study, our objective was, on the global scale, to reveal the effects of geological influences, modern climate and climate stability on the diversity, endemism and morphological innovations of genus *Saussurea*, the role of phylogenetic conservatism in the distribution pattern of the species and the contribution of morphological innovation to diversity. Specifically, we addressed two questions: (i) what are the present diversity and endemism patterns of the *Saussurea* species, and the underlying driving mechanisms? and (ii) what are the present diversity and endemism patterns of the specialized morphological innovations in this genus and the underlying driving mechanisms? Answering these questions on a global scale can provide insights for understanding the distribution pattern, speciation and morphological innovations of mountain species.

## Materials and Methods

### Species distribution data

Distribution records were collected from published floras, online databases, herbarium specimens, research papers and monographs **[see**Supporting Information 1**]**. We adopted the taxonomic classification of *Saussurea* according to Chen (2015) and Raab-Straube (2017). Records providing coordinates of species occurrences account for a large proportion of the data, e.g. ca. 70 % GBIF records (GBIF Occurrence Download 10.15468/dl.teopcs, 2019-10-11), specimens collected in the last two decades, records in the recent floras and monographs. To ensure the maximum use of effective/correct records, we deleted incorrect records based on three methods. First, we used algorithmic detection based on R package ‘CoordinateCleaner’ (Zizka 2018) to identify outlier coordinates, zero coordinates, identical latitude/longitude and invalid coordinates. Second, we collected a large number of identification records from authoritative monographs **[see**Supporting Information 1**]** and experts of this genus to ensure the accurate data sources, e.g. Eckhard von Raab-Straube (Botanic Garden and Botanical Museum Berlin), Yousheng Chen (Chinese Academy of Sciences) and so on. Third, we manually filtered the data based on the distribution information (e.g. altitude, habitat) from expert identifications and monographs, and deleted these error records. The species distributions recorded at the level of specific location (e.g. villages, towns, counties, peaks, nature reserves) were georeferenced into coordinates. To eliminate the influence of area on the estimation of biodiversity, the species distribution data were transferred into 1° × 1° grid cells. The grid size was chosen on the basis of the following rules. First, 1° × 1° grid cell is the ‘finest spatial resolution that is appropriate for this broad-scale analysis’ (Zuloaga *et al.* 2019) and this scale was also widely used and verified in large-scale spatial analyses (Wang *et al.* 2017; Antonelli *et al.* 2018; Lu *et al.* 2018; Shrestha *et al.* 2018). Second, it is suitable to select 1 degree in this study judging from the data/records type of and the distribution pattern of *Saussurea*. *Saussurea* species rarely form foundation species, so their distributions are always sporadic. Therefore, finer resolutions would highlight the fragmentation of the distribution of *Saussurea* and dilute the effects of the diversity indexes and environmental indicators in the analysis. We also analysed the distribution patterns based on 0.5° × 0.5° grid cell **[see**Supporting Information 1**]**, which show similar diversity patterns but fragmented connectivity patterns, compared with these based on 1° × 1° grid cell. The final distribution data included 466 species of *Saussurea***[see****Supporting Information 2****]**. Moreover, we also compiled a distribution database for 120 species with any special morphological trait (SMT), including greenhouse, woolly, cushion and stemless. Greenhouse species are those with the capitula or inflorescence enclosed, half-enclosed or subtended by coloured (yellowish, red or purple-black) bracts, mainly including ‘snow lotus’ in subg. *Amphilaena*. Woolly species are those with dense hairs (lanate, villous, sericeous or tomentose), mainly including ‘snow rabbit’ in subg. *Eriocoryne* and other species with dense hairs. Cushion species are those with the dense branching, forming a compact canopy. Stemless species are those with stemless or rosette leaves that grow close to the ground.

### Environmental variables and spatial indices

#### Geological influences.

(i) Alpine plants developed specific mechanisms adapting them to high-altitude environments (Sun *et al.* 2014), so we calculated the average altitude (Alt) within the grid as a variable reflecting this indicator. (ii) We calculated the standard deviation of altitude (Alt_SD) in a grid cell to reflect the habitat heterogeneity (Shrestha *et al.* 2018) or topographic uplift (Yu *et al.* 2019). The altitude data layer was downloaded from National Oceanic and Atmospheric Administration (https://www.ngdc.noaa.gov) with a 30-arc-second resolution.

#### Modern climate.

Modern climate is the average for the years 1970–2000 (Hijmans *et al.* 2005), determines the availability of energy and water and is considered an important factor affecting the distribution of plants (Currie *et al.* 2004). We used the modern mean annual temperature (MAT) and the modern mean annual precipitation (MAP) to reflect the modern climate.

#### Climate stability.

Climate stability has an important impact on local biodiversity, especially for species with poorer dispersal ability (Sandel *et al.* 2011). (i) A value to indicate climatic anomaly was calculated as modern MAT/MAP minus the corresponding value at the Last Glacial Maximum (LGM), i.e. MAT_ano and MAP_ano. (ii) Climate change velocity (Vel) is a measure of the local rate of change in the climate conditions (Loarie *et al.* 2009), and this was calculated according to Sandel *et al.* (2011) based on modern and LGM MAT. The data layer for LGM temperature was obtained from the mean values of the CCSM3 (Otto-Bliesner *et al.* 2006) and MIROC-ESM (Hasumi and Emori 2004) models. Bioclimatic variables were downloaded from the WorldClim database (Hijmans *et al.* 2005, http://www.worldclim.org).

Species richness (SR) and weighted endemism (WE) were used to reflect the diversity and endemism in a grid cell for all *Saussurea* and *Saussurea* with SMTs. Species richness was calculated as the number of the total species in a grid cell. Weighted endemism emphasized cells with high rates of restricted species and was calculated as ‘the sum of the reciprocal of the total number of cells in which each species is found’ (Linder 2001).


$$\begin{array}{l} WE=\sum_{S}\frac{1}{R_{S}} \end{array}$$

where S is all the species found in a grid cell; and *R*_S_ is the range in which this species occurs. The calculations of SR and WE were carried out in Biodiverse V2.0 (Laffan *et al.* 2010).

### Phylogenetic analyses

A well-supported phylogenetic dating framework based on whole chloroplast genomes for *Saussurea* was obtained from Xu *et al.* (2019b); this is the most reliable phylogeny available and includes 125 *Saussurea* species. The phylogeny of *Saussurea* in this study includes 125 species and misses ~335 species. To assess the impact of phylogenetic uncertainty, we propose here a novel analytical strategy (for details, **see**Supporting Information 1). The environmental variables (Alt, Alt_SD, MAT, MAP, MAT_ano, MAP_ano, Vel) for each species were calculated by putting every occurrence point into its 0.25° × 0.25° grid cell and calculating the mean values of all grid cells. Blomberg’s *K* is used to compare the observed value of each variable with that of the predicted value based on the Brownian motion model (Blomberg *et al.* 2003). Although Blomberg’s *K* discriminates poorly between more complex models of trait evolution, it allows to detect subtle changes in phylogenetic signal and is insensitive to sample size (Münkemüller *et al.* 2012), which is suitable in this study. A *K*-value close to 1 indicates that the evolutionary process is close to Brownian motion, i.e. there is a certain degree of phylogenetic signal or of conservatism. *K* close to 0 indicates that evolution tends to be random, and *K* > 1 indicates that traits are conservative. Blomberg’s *K* was calculated using the package ‘phylosignal’ (Keck *et al.* 2016) in R (R Core Team 2018) based on the phylogenetic tree and the environmental variables matrix. Ancestral character estimation was conducted using the ‘ape’ package based on two methods: Felsenstein’s phylogenetic independent contrasts (PIC) and residual maximum likelihood (REML) (Paradis and Schliep 2019). The PIC method is a Brownian motion-based algorithm, but takes only descendants of each node into account when estimating ancestral character. The REML method first calculates the ancestral value at the root, then the variance of the Brownian motion process is estimated by optimizing the residual log-likelihood. These two methods are frequently used estimate the ancestral niches (Shrestha *et al.* 2018). In order to ensure the stability of the results, we compare the results based on different methods.

### Statistical analyses

Spatial simultaneous autoregressive (SAR) error models and modified *t*-tests were run to account for spatial autocorrelation using the MuMIn (Bartoń 2019), SpatialPack (Osorio and Vallejos 2019) and spdep (Bivand *et al.* 2018) packages in R. First, we conducted ordinary least squares (OLS) linear regressions and SAR to explore bivariate relationships between SR, WE and each variable. We then constructed multiple regression models and selected the best model based on Akaike’s information criterion (AIC) and calculated model-averaged coefficients for the predictors based on AIC weights of the models. The sum of AIC weights in all models for each predictor was calculated to reflect the statistical support. In the global SAR model, we divided environmental variables into two groups due to the strong collinearity between Alt_SD and Alt (0.794; **see**Supporting Information 1**—Table S1**): (a) Alt_SD + MAT + MAP + MAT_ano + MAP_ano + Vel; (b) Alt + MAT + MAP + MAT_ano + MAP_ano + Vel. In the model for SMT species, we divided environmental variables into two groups due to the strong collinearity between Alt_SD and Vel (−0.88; **see**Supporting Information 1**—Table S2**): (c) Alt_SD + Alt + MAT + MAP + MAT_ano + MAP_ano; (d) Alt + MAT + MAP + MAT_ano + MAP_ano + Vel. We also used a modified *t*-test to explore the relationships between SR/WE of species with SMTs and total SR/WE of *Saussurea* in a grid cell. To assess the impact of sampling bias on our results, we used Oliveira *et al.*’s (2017) methods to remove any cells with sampling bias **[see**Supporting Information 1**]**.

To further quantify the independent and combined effects of geological influences, modern climate and climate stability on diversity and endemism, we conducted a partial regression analysis using the ‘vegan’ package (Oksanen *et al.* 2015) in R, because it can contain collinear variables prior to partitioning. All seven environmental variables were assigned into one of three groups of factors: geological influences, modern climate, climate stability, for which we were able to obtain the independent explained variance, shared explained variance and totally explained variance.

## Results

**Figure 1.**
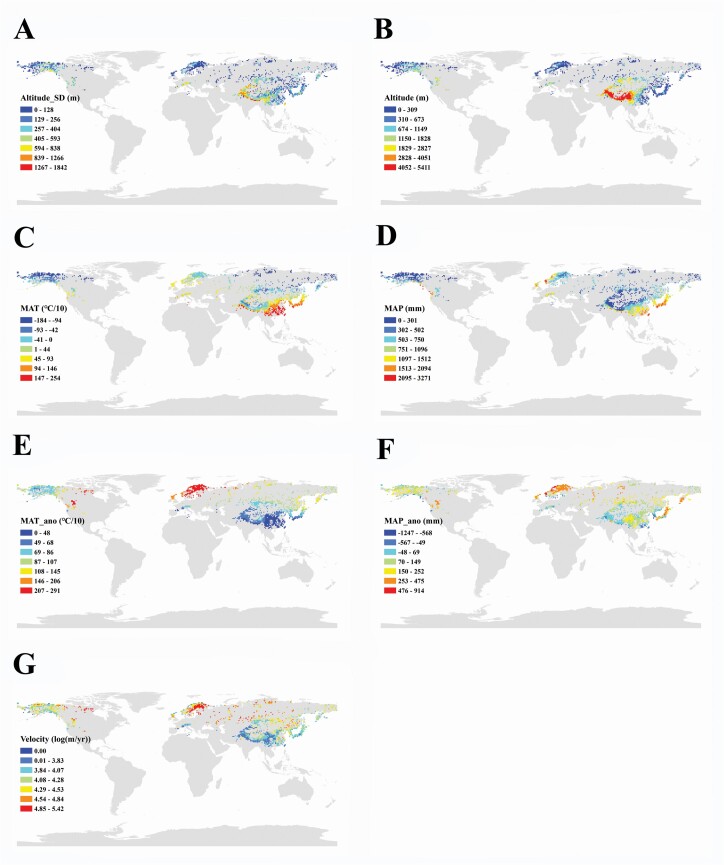
Spatial distribution of predictors in 1° × 1° grid cells based on *Saussurea* globally. (A) Alt_SD: standard deviation of altitude; (B) Alt: mean altitude; (C) MAT: modern mean annual temperature; (D) MAP: modern mean annual precipitation; (E) MAT_ano: modern mean annual temperature anomaly; (F) MAP_ano: modern mean annual precipitation anomaly; (G) Vel: climate change velocity.

**Figure 2.**
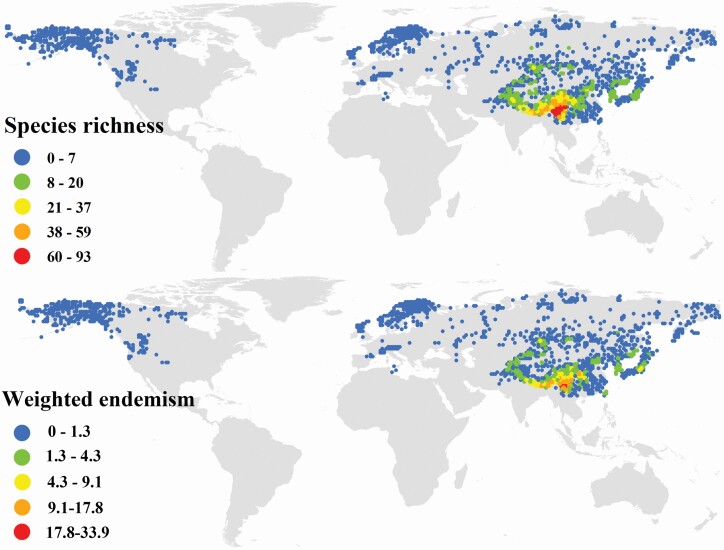
Global distributions of species richness (SR) and weighted endemism (WE) for *Saussurea*.

**Figure 3.**
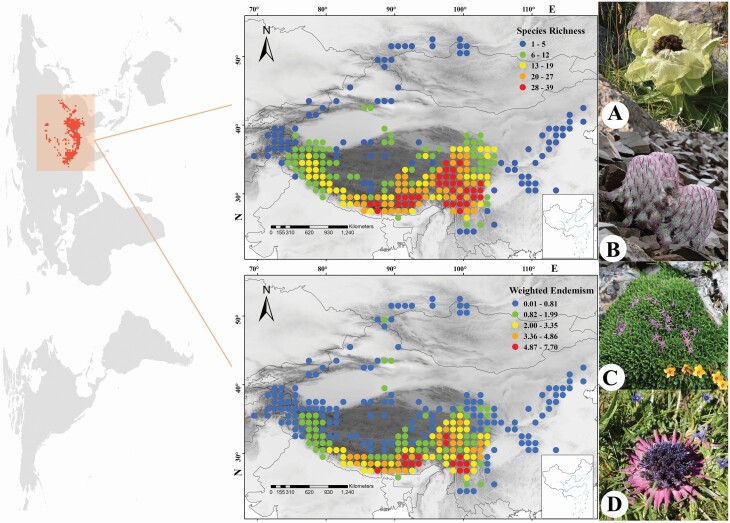
Spatial distributions of species richness (SR) and weighted endemism (WE) for species with special morphological traits in the genus *Saussurea*. (A) Greenhouse (*S. involucrata*); (B) woolly (*S. medusa*); (C) cushion (*S. subulata*); (D) stemless (*S. stella*). (A) By Z. Z. Yang, (B–D) by Y. Z. Zhang. Grey shading indicates altitudes, the lower right corner of maps is the boundary line of China.

The spatial patterns of environmental variables are shown in Fig. 1A–G. In brief, the highest values of Alt_SD and Alt were mostly found in the QTP (especially in the HDM and Himalayas); the highest values of MAT and MAP were mostly found in S China and SW Japan; the highest values of climate stability variables were mostly found in the high latitudes in the northern hemisphere, i.e. N Europe, N America and the Far East, while the QTP and its surrounding regions had lower climate stabilities. For global *Saussurea* and SMT species, the HDM and eastern Himalayas host the highest SR and endemism (the SMT species only occur in E Asia), while N Europe, N America and the Far East host the lowest SR and endemism (Figs. 2 and 3).

**Figure 4.**
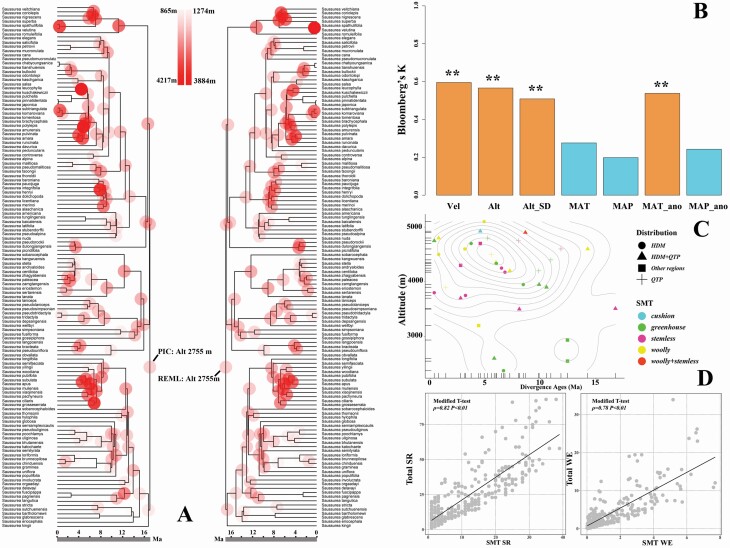
Results of phylogenetic analyses and modified *t*-tests. (A) Ancestral character estimation of altitude based on two methods: PIC (left) and REML (right). The two methods generated consistent results at root; (B) phylogenetic signals for environmental variables assessed based on Bloomberg’s *K*. **P < 0.01. Vel: climate change velocity; Alt: mean altitude; Alt_SD: standard deviation of altitude; MAT: mean annual temperature; MAP: mean annual precipitation; MAT_ano: mean annual temperature anomaly; MAP_ano: mean annual precipitation anomaly; (C) distributions of divergence ages and altitude for species with special morphological traits. Contour lines represent kernel density estimates. Scales on the axes represented rug lines. HDM: the Hengduan Mountains; QTP: the Qinghai–Tibet Plateau. (D) Pearson’s correlation between SR/WE of species with special morphological traits and total SR/WE of *Saussurea* based on modified *t*-test. *ρ*: correlation coefficient.

The reconstructed ancestral altitude niche based on two different methods (PIC and REML) generated consistent results at root (ca. 17 Ma), i.e. *Saussurea* species originated at ~2755 m (Fig. 4A). In the current HDM, the root altitude is just in the intermediate elevation zone (Fig. 1B). Phylogenetic signal analyses indicated that Alt_SD, Alt, MAT_ano and Vel exhibited a certain degree of phylogenetic signalling or conservatism (0.5 < *K* < 1, *P* < 0.01; Fig. 4B). The scatter plot of altitude and divergence age indicated the SMTs generally originated at high altitudes (4000–5000 m) during recent historical periods (concentrated between ca. 4 and 8 Ma) (Fig. 4C). Due to incomplete sampling of the dating phylogeny, the lack of sister species between some nodes may lead to older estimates of the divergence ages, but the figure still reflects the general divergence trend.

In conclusion, Alt, Alt_SD, MAT_ano and Vel always have constantly strong predictive powers (reflected by a significant *P*-value, high coefficient, the best model and the highest AIC weighting) in all models for SR, WE in both global and SMT species analyses, including single variable regression models, multi-predictor regression models and those models taking into account sampling bias (Tables 1 and 2; **see****Supporting Information 1—Tables S3****–****S5**), i.e. Alt_SD and Alt, representing geological influences, had positive effects on diversity and endemism; MAT_ano and Vel, representing climate stability, had negative effects on diversity and endemism. SR/WE of species with SMTs and total SR/WE of *Saussurea* exhibited significantly high correlations (*ρ* = 0.82, *P* < 0.01; *ρ* = 0.78, *P* < 0.01; Fig. 4D).

**Table 1.** Results from multi-predictor SAR models of global distributions. SR: species richness; WE: weighted endemism; Vel: climate change velocity; Alt: mean altitude; Alt_SD: standard deviation of altitude; MAT: mean annual temperature; MAP: mean annual precipitation; MAT_ano: mean annual temperature anomaly; MAP_ano: mean annual precipitation anomaly. Coef = coefficients of models with the highest AIC weights, *w*1 = AIC weights of the best model, *w* = summed AIC weights of all models containing that variable, Coef_ave = averaged standardized regression coefficients, *R*^2^ = Nagelkerke pseudo-*R*^2^. (a) model: Alt_SD + MAT + MAP + MAT_ano + MAP_ano + Vel; (b) model: Alt + MAT + MAP + MAT_ano + MAP_ano + Vel. **P < 0.01, ***P < 0.001.

(a)	SR			WE		
	Coef	*w*	Coef_ave	Coef	*w*	Coef_ave
Alt_SD	0.103***	1	0.103***	0.126***	1	0.128***
MAT	—	0.25	−0.002	—	0.44	0.019
MAP	—	0.27	−0.004	—	0.27	−0.001
MAT_ano	−0.233***	1	−0.239***	−0.336***	1	−0.337***
MAP_ano	—	0.35	0.003	—	0.41	0.005
Vel	−0.064**	1	−0.065**	−0.062***	1	−0.063**
*R* ^2^	0.824			0.820		
*w*1	0.336			0.239		
(b)	SR			WE		
	Coef	*w*	Coef_ave	Coef	*w*	Coef_ave
Alt	0.168***	1	0.162***	0.190***	1	0.189***
MAT	0.052	0.52	0.026	0.107**	1	0.106**
MAP	—	0.27	0.001	—	0.30	0.005
MAT_ano	−0.231***	1	−0.243***	−0.302***	1	−0.306***
MAP_ano	—	0.36	0.003	—	0.39	0.004
Vel	−0.092***	1	−0.092***	−0.104***	1	−0.103***
*R* ^2^	0.826			0.822		
*w*1	0.243			0.433		

**Table 2.** Results from multi-predictor SAR models of SMTs distributions. SR: species richness; WE: weighted endemism; Vel: climate change velocity; Alt: mean altitude; Alt_SD: standard deviation of altitude; MAT: mean annual temperature; MAP: mean annual precipitation; MAT_ano: mean annual temperature anomaly; MAP_ano: mean annual precipitation anomaly. Coef = coefficients of models with the highest AIC weights, *w*1 = AIC weights of the best model, *w* = summed AIC weights of all models containing that variable, Coef_ave = averaged standardized regression coefficients, *R*^2^ = Nagelkerke pseudo-*R*^2^. (c) model: Alt_SD + Alt + MAT + MAP + MAT_ano + MAP_ano; (d) model: Alt + MAT + MAP + MAT_ano + MAP_ano + Vel. *P < 0.05, **P < 0.01, ***P < 0.001.

(c)	SR			WE		
	Coef	*w*	Coef_ave	Coef	*w*	Coef_ave
Alt_SD	0.143***	1	0.132***	0.211***	1	0.206***
MAT	—	0.39	0.022	—	0.15	0.001
MAP	−0.15*	0.81	−0.118	−0.203*	0.9	−0.183
MAT_ano	—	0.17	−0.001	—	0.15	−0.001
MAP_ano	—	17	−0.002	—	0.16	−0.002
Alt	0.179***	1	0.200***	0.143**	1	0.148**
*R* ^2^	0.791			0.684		
*w*1	0.297			0.435		
(d)	SR			WE		
	Coef	*w*	Coef_ave	Coef	*w*	Coef_ave
Vel	−0.127***	0.96	−0.102*	−0.177***	1	−0.165**
MAT	—	0.47	0.034	—	0.23	0.005
MAP	−0.127	0.57	−0.071	−0.161	0.68	−0.112
MAT_ano	—	0.23	−0.002	—	0.24	−0.004
MAP_ano	—	0.23	−0.004	—	0.23	−0.004
Alt	0.195***	1	0.232***	0.170**	1	0.188**
*R* ^2^	0.787			0.674		
*w*1	0.18			0.257		

In global partial regression analyses for diversity and endemism **[see****Supporting Information 1—Table S6****]**, geological influences independently accounted for more variance than any other factors (0.14–0.23). There are also strong intersections between geological influences and climate stability (0.15–0.18). Climate stability was the second strongest explanatory factor. In partial regression analyses for SMT diversity and endemism **[see****Supporting Information 1—Table S6****]**, geological influences independently accounted for more variance than any other factors (0.22–0.40) and modern climate and climate stability had similar explanatory powers.

## Discussion

### Diversity and endemism of *Saussurea*

Previous studies suggested that alpine plant genera on the QTP originated from various regions, for instance, *Solms-laubachia* and *Juniperus* originated from central Asia (Yue *et al.* 2009; Mao *et al.* 2010); *Diapensia* and *Cassiope* originated from high-latitude regions (Hou *et al.* 2016a, b); *Draba* originated from the northern QTP (Chen *et al.* 2010); and *Lagotis* (Li *et al.* 2014), *Gentiana* (Favre *et al.* 2016) and *Saussurea* (Xu *et al.* 2019b) originated locally on the QTP. What is interesting is that, no matter where they originated, many genera diversified on the QTP or even formed diversity or endemism centres in this region. Our results suggest that *Saussurea* is mainly found on the QTP, particularly in the HDM region, and that the QTP served as the diversity and endemism centre of this genus. Moreover, geological influences (the average altitude and the standard deviation of altitude) and climate stability (the climatic anomaly of MAT and the climate change velocity) played an important role in driving *Saussurea* diversity and endemism.

The average altitude and the standard deviation of altitude, to some extent, are associated with and thus can reflect the intensity of mountain uplift/orogeny (Yu *et al.* 2019). They are, therefore, regarded as important factors promoting the biodiversity of mountains, because orogeny can greatly shape diverse topography, heterogeneous climatic types and long-term climatic stability (Hoorn *et al.* 2013; Wen *et al.* 2014; Xing and Ree 2017). We think that the recent and drastic orogeny in the QTP, mainly in the Himalaya and HDM subregions (see review in Muellner-Riehl 2019), made this region the diversity centre of *Saussurea*. Phylogenetic analyses also suggest that *Saussurea* originated at 2755 m in the HDM during the middle Miocene. The orogenic history of the HDM has been inferred to have occurred between the late Miocene and late Pliocene (Xing and Ree 2017 and references therein). This timeline also supports the suggestion that orogeny may have contributed to the diversification of *Saussurea*. Moreover, the standard deviation of altitude (or altitude range) also reflects local habitat heterogeneity and availability induced by orogeny, which can provide more ecological niches to aid diversification and promote speciation (Wang *et al.* 2017; Shrestha *et al.* 2018). Thus, we further conclude that the difference in habitat heterogeneity across the northern hemisphere (Fig. 1B) also shaped the current diversity pattern of *Saussurea*. Topographic heterogeneity, which means various and available habitats, can provide specialized habitat requirements to a range of narrowly endemic species (Crisp *et al.* 2001), resulting in the QTP and surrounding regions supporting higher endemism of *Saussurea*. Moreover, exactly as described by the term ‘sky islands’, the alpine flora is often isolated by deep valleys, with the result that ‘lots of species are endemic to specific mountain peaks’ (Xu *et al.* 2014; Luo *et al.* 2016; Sun *et al.* 2017), which also promotes higher endemism in these areas with intense isolation, e.g. the HDM and Himalayas.

Furthermore, our results indicate that the relatively stable glacial–interglacial climate environment in the QTP positively drove the diversity and endemism of *Saussurea*, whilst in North America, Europe and the Far East with drastic climate fluctuations there was less diversity and endemism. The ice coverage in the Quaternary dramatically changed patterns of global biodiversity, leading to massive extinctions of terrestrial biota, particularly in mid- to high-latitude areas (Webb and Bartlein 1992; Fig. 1E–G). Many mountain systems in lower latitudes acted as refugia during the ice ages and thus produced abundant biodiversity (Wallis *et al.* 2016). Glaciations of mountains in lower latitudes could result in vicariance and thus promote alpine speciation (Wallis *et al.* 2016 and references therein). In addition, glaciations can also form a ‘flickering connectivity system’, with dynamic changes in habitat connectivity thus permitting intermittent gene flow that significantly drives speciation (Flantua *et al.* 2019; Muellner-Riehl 2019). Quaternary glacial–interglacial climate changes also had important effects in shaping the distribution pattern of endemic species (Feng *et al.* 2016). The unstable glacial–interglacial climate reduced endemism as a result of increased extinction and reduced speciation (Feng *et al.* 2019). Moreover, dispersal limitation also greatly affected the endemic pattern during paleoclimatic fluctuations, especially for species less able to migrate (Sandel *et al.* 2011). Consistent with previous results, we found that areas with smaller paleoclimatic fluctuations harbour more endemic species.

In addition, species are adapted to ancestral niches, so that the environment away from the ancestral niches is not conducive to survival (Xu *et al.* 2019a). The phylogenetic results indicate that *Saussurea* originated at high altitude, and geological influences (the average altitude and the standard deviation of altitude) and climate stability (the climatic anomaly of MAT and the climate change velocity) exhibited a certain degree of conservatism. We argue that the conservatism of the ancestral niches led to a decrease in species diversity as the ‘out of Tibet’ process occurred. The environmental features shaped by high altitudes, such as temperature, are sometimes reproduced at latitude, but not always, as is the case for intense radiation, low atmospheric pressure, irregular rainfall, etc. (Körner 2003). Therefore, species originating at high altitudes may also not be able to adapt during the process of migration to higher latitudes. In general, geological influences and climate stability have, acting in a concert, shaped the distribution pattern of the *Saussurea* species in the northern hemisphere at both ecological level and phylogenetic level.

### Morphological innovations and adaptive evolution

It has been suggested that morphological specialization is commonly associated with high species diversity (e.g. Armbruster and Muchhala 2008 and references therein). However, previous studies mainly focused on specialized flowers adapted to particular pollinators, thus increasing reproductive isolation and in turn increasing speciation rates (see review in Rieseberg and Willis 2007). Some studies have revealed mechanisms driving specialized morphological traits adapted to the severe alpine environments (Sun *et al.* 2014 and references therein); however, few studies have attempted to find associations between the distributions of specialized morphological innovations and environmental factors at a large scale. Our results show that geological factors and climate stability are significantly associated with morphological innovations: areas with higher altitude, higher altitude heterogeneity and smaller climate changes harbour high diversity and endemism with specialized morphological traits (Table 2; Fig. 3). We think that, because geological history and glacial–interglacial climate changes have greatly altered the local environmental conditions, for alpine plants in particular, the survival conditions have become especially extreme, including lower temperatures, poorer soils, lower atmospheric pressures and stronger radiation (Körner 2003; Nagy and Grabherr 2009; Sun *et al.* 2014). The specialized morphological traits evolved as adaptations to the severe environments encountered in alpine regions (Song *et al.* 2013; Chen *et al.* 2015, 2019). Our results indicate that species with SMTs are mainly found on the QTP and its surrounding areas. The limited distribution range of specialized morphological species may be explained by the spatial distribution patterns of geological conditions and paleoclimatic changes, i.e. such species are found in areas with suitable environments (e.g. high altitude; Fig. 4C) and relatively stable climate. Thus, we argue that the profound orogeny and tolerable climate changes in the QTP did, indeed, promote the morphological innovations which further facilitated speciation and endemism of *Saussurea* in this region. The strong relationships between SMTs and local diversity and endemism also suggest that foliar morphological innovations are an important process of diversification in *Saussurea* (Fig. 4D).

## Conclusions

To study alpine speciation and adaptive evolution, *Saussurea* was an ideal subject: originating in the middle altitudes of the east QTP, diffusing to lower and higher altitudes, associated with high mountains in the northern hemisphere and harbouring amazing morphological innovations (Raab-Straube 2017; Xu *et al.* 2019b). Although some results in this study have been mentioned in previous studies (Wang *et al.* 2009; Xu *et al*. 2019b), they mainly focused on systematic and biogeographic problems, not global diversity and endemism patterns. They also did not derive detailed conclusions based on a combination of spatial statistics and phylogenetic analyses. This study indicates that high altitudes had positive effects on diversification and endemism of this genus, and could also provide appropriate environmental pressures leading to the formation of morphological innovations. In addition, high topographic heterogeneity provided more habitats, allowing more species to occupy different ecological niches, further facilitating diversity and endemism. Moreover, small glacial–interglacial climate changes provided stable conditions for increasing speciation, promoting endemism and reducing extinction. All these components together made the QTP a diversity and endemism centre for *Saussurea* species. We also cannot ignore the important role of phylogenetic conservatism in the distribution of alpine species, because alpine species prefer extreme environments and are less adaptable to new ones. Specialized morphological traits were evolved to allow survival in severe alpine environments induced by geological influences and paleoclimate changes, and thus these played important roles in alpine speciation and adaptive evolution. In summary, this study offered an excellent example to study and understand the diversity pattern, endemism pattern and adaptive evolution of alpine species in the northern hemisphere.

 However, the incomplete sampling of *Saussurea* phylogeny may, to some extent, affect our results and restrict further analyses (e.g. speciation rate, extinction rate and so on). To eliminate such potential effect, we first compared spatial and phylogenetic results to verify the consistency and robustness of our conclusions. Then, we constructed a null model to assess the potential impact of phylogenetic uncertainty, which implies that the simulated phylogenetic uncertainty analysis does not meet the ideal test requirements until a nearly complete phylogeny is constructed. But the results of null model also support our spatial and phylogenetic results. Our study provides a meaningful attempt to explore phylogenetic uncertainties in a species-rich genus. We believe that future studies can further explain the speciation and evolution of *Saussurea* based on a well-sampled phylogeny. Moreover, our research has not yet revealed the specific adaptative mechanisms for SMTs, which requires genomic approaches. Here, we hope to point out directions for future in-depth researches.

## Supporting Information

The following additional information is available in the online version of this article—

Supporting Information 1. Supplementary methods and results.

Supporting Information 2. The Saussurea species checklist.

Supporting Information 3. Data and codes used in this study (include files of Supplementary_Materials_S4–7).

plab018_suppl_Supplementary_Materials

## Data Availability

All data generated and analysed during this study are obtained from open database as showed in Materials and Methods section (include raw data) and its Supporting Information files: Supporting Information 1 (Supplementary methods and results), Supporting Information 2 (The *Saussurea* species checklist). The software and calculation process were described in the Materials and Methods section. Data and codes used in this study are available in Supporting Information 3.

## Notes

**Evolution & Diversity. Chief Editor:** Jeremy Beaulieu
