# Supplementary material for: Alpine speciation and morphological innovations: revelations from a species-rich genus in the northern hemisphere
Source: AoB Plants. 2021 Apr 15;13(3):plab018. doi: 10.1093/aobpla/plab018 (PMC8129467; doi:10.1093/aobpla/plab018)
Supplement: plab018_suppl_Supplementary_Materials [file plab018_suppl_supplementary_materials.zip › plab018_suppl_Supporting Information 1.docx]

**Supplementary Information 1.** **Supplementary methods and results**

**Data sources for global *Saussurea* distributions**

The *Saussurea* distribution records were collected based on the following sources:

Published floras: *Flora of China*, *Flora Reipublicae Popularis Sinicae*, *Flora of Pan-Himalaya*, *Vascular plants of the Hengduan Mountains*, *Annotated Checklist of the Flowering Plants of Nepal*, *Flora of Pakistan*, *Flora of Bhutan*, *Flora of Japan*, *Flora of North America*. The distribution data for *Saussurea* in China were also from provincial and local floras, e.g. *Flora of Tibet*, *Flora of Yunnan*, *Flora of Qinghai* and so on.

Online databases: National Specimens Information Infrastructure (NSII) (http://www.nsii.org.cn/), Chinese Virtual Herbarium (http://www.cvh.ac.cn/), JSTOR (https://plants.jstor.org/), Global Compositae Database (http://www.compositae.org/), GBIF (https://www.gbif.org/), eFloras (http://www.efloras.org/), Virtual Guide to the Flora of Mongolia (https://floragreif.uni-greifswald.de/), The Euro+Med PlantBase (http://ww2.bgbm.org/EuroPlusMed/), National Depository Bank of Live Systems (https://plant.depo.msu.ru/).

Monographs: Shi and Raab-Straube (2011), Chen (2015), Raab-Straube (2017).

Research papers: Chen and Wang (2018), Rana et al. (2018), Zhang et al. (2019), Xu et al. (2019). Some papers including species that had been recorded in floras and monographs are not cited.

**Sampling bias**

We used Oliveira et al.’s (2017) method to calculate the predicted species richness based on observed species richness and records of species distribution. Then, the ratio of observed species richness/predicted species richness was used as a measure of completeness of each grid cell. Those cells with a completeness value < 70% (Sánchez-Fernández et al. 2008) were considered to have been inadequately sampled (**Figure S1**). For global regression models, we removed 42% of grid cells (650/1543) before analyses. We maintain the SMT analyses without removing grid cells, because there was only one grid cell with inadequate sampling. Our results suggest that the relationships between SR, WE and variables were stable, i.e. Alt, Alt_SD, MAT_ano and Vel always exhibited strong predictive powers in the different models (**Table 1, Table 2, Table S3, Table S4 and Table S5**).

**Phylogenetic uncertainty**

Phylogenetic uncertainty has attracted more and more attention of ecologists and biogeographers in recent years. It may arise from two main sources, i.e. multiple alternative topologies resulting from weak, missing, or conflicting phylogenetic support and incomplete or unrepresentative sampling of known species (Rangel et al. 2015). Our phylogeny obtained from Xu et al. (2019), constructing based on credible sampling and whole chloroplast genomes, show stable phylogenetic support. Therefore, we mainly focus on the second source of phylogenetic uncertainty derived from missing species. The phylogeny of *Saussurea* in this study includes 125 species and misses about 335 species. To assess the impact of phylogenetic uncertainty, we randomly inserted missing species into the partially known tree based on SUNPLIN method (Martins et al. 2013). We calculated the null model distributions (1000 iterations) and used Z values of Bloomberg’s K to evaluate the difference between the observed values and the null models. Z value was calculated as: Z-value = (the observed value - the mean value of null model) / the standard deviation of null model. Simulations with uncertainty for phylogenetic investigation reveal that the observed K value is much larger than the null models: Z (mean altitude) = 253.853, Z (standard deviation of altitude) =187.8866, Z (mean annual temperature) = 121.0524, Z (mean annual precipitation) = 70.66105, Z (mean annual temperature anomaly) = 167.0218, Z (mean annual precipitation anomaly) = 82.91225 and Z (climate change velocity) = 244.0236. Such extremely robust results imply that the simulated phylogenetic uncertainty analysis does not meet the ideal test requirements until a nearly complete phylogeny is constructed. But the distinct high values of mean altitude, standard deviation of altitude, mean annual temperature anomaly and climate change velocity also strongly support our results about geological influences and climate stability, which makes our results more credible. In fact, phylogenetic uncertainty evaluation has always been a difficulty in many phylogenetic analysis of species-rich genera due to a high proportion of incomplete phylogenetic sampling. Therefore, it is also understandable that some studies have chosen not to explore phylogenetic uncertainties (Shrestha et al. 2018; Xu X et al. 2019). Our study provides a meaningful attempt. Moreover, comparative analyses were used in our study, i.e. the two results from spatial analyses in global scale and phylogenetic analyses support each other, which also provides robust support for our conclusions.

**Figure S1. Assessment of sampling completeness. Red indicates those well-surveyed grid cells (completeness value** **≥ 70%); Blue indicates those inadequately surveyed grid cells (completeness value < 70%).**


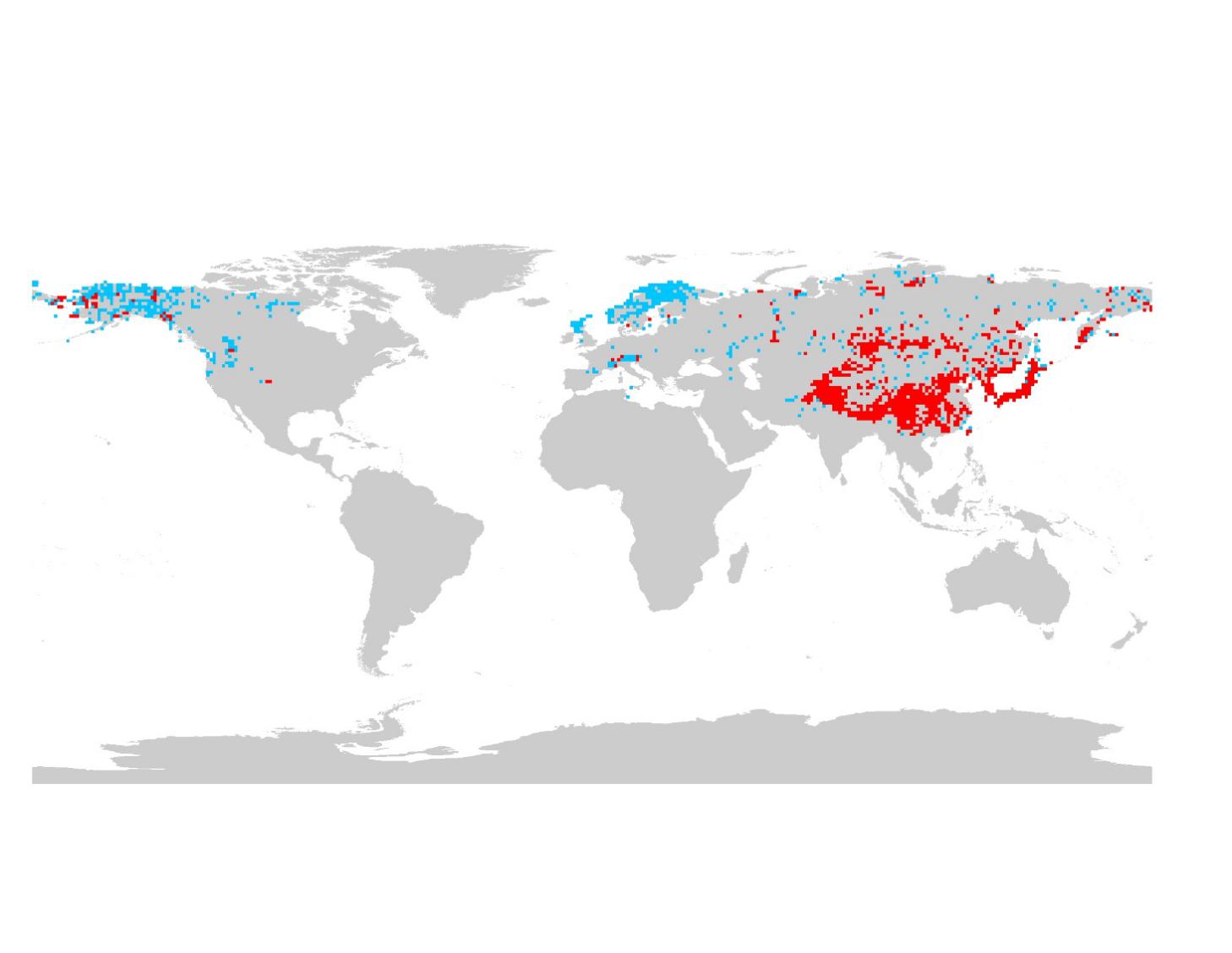


Figure S2. Global distributions of species richness (SR) in 0.5°×0.5°.


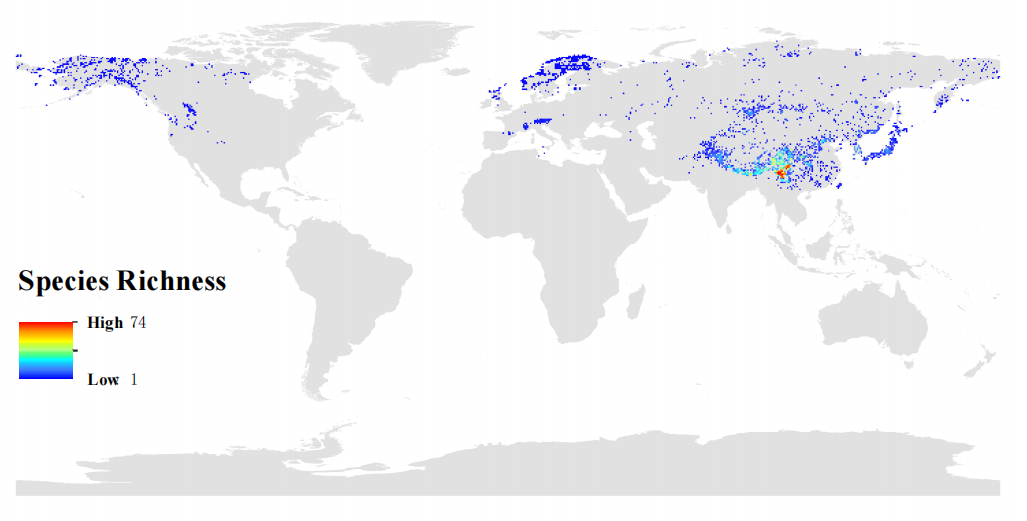


Figure S3. SMT distributions of species richness (SR) in 0.5°×0.5°.


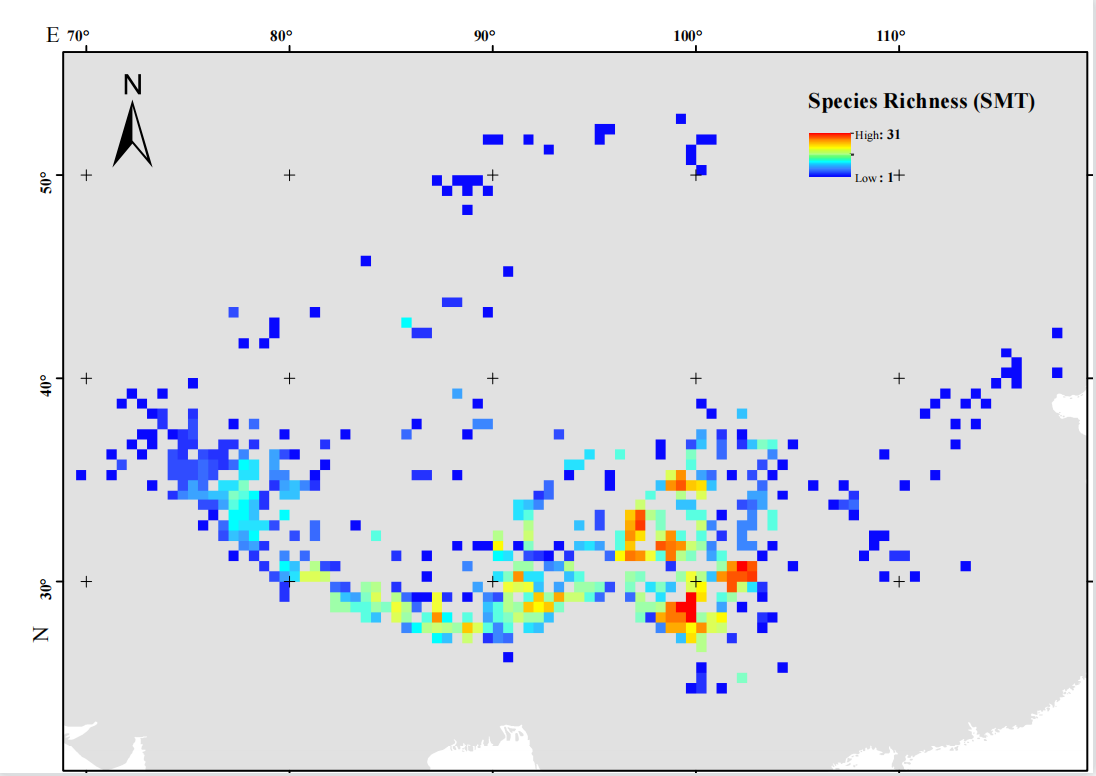


**Table S1. Correlation coefficients between different environmental variables in the global model. Red indicates strong collinearity. Vel: climate change velocity; Alt: mean altitude; Alt_SD: standard deviation of altitude; MAT: mean annual temperature; MAP: mean annual precipitation; MAT_ano: MAT anomaly; MAP_ano: MAP anomaly.**

|  | Alt | Alt_SD | MAT | MAP | MAT_ano | MAP_ano | Vel_vo |
| --- | --- | --- | --- | --- | --- | --- | --- |
| Alt | 1.00 | 0.79 | 0.11 | -0.14 | -0.43 | -0.07 | -0.50 |
| Alt_SD | 0.79 | 1.00 | 0.18 | 0.12 | -0.44 | -0.08 | -0.71 |
| MAT | 0.11 | 0.18 | 1.00 | 0.51 | -0.34 | -0.05 | -0.28 |
| MAP | -0.14 | 0.12 | 0.51 | 1.00 | -0.06 | 0.02 | -0.15 |
| MAT_ano | -0.43 | -0.44 | -0.34 | -0.06 | 1.00 | 0.21 | 0.67 |
| MAP_ano | -0.07 | -0.08 | -0.05 | 0.02 | 0.21 | 1.00 | 0.13 |
| Vel | -0.50 | -0.71 | -0.28 | -0.15 | 0.67 | 0.13 | 1.00 |

**Table S2. Correlation coefficients between different environmental variables in the SMT model. Red indicates strong collinearity. Vel: climate change velocity; Alt: mean altitude; Alt_SD: standard deviation of altitude; MAT: mean annual temperature; MAP: mean annual precipitation; MAT_ano: MAT anomaly; MAP_ano: MAP anomaly.**

|  | Alt | Alt_SD | MAP | MAT | MAT_ano | MAP_ano | Vel_vo |
| --- | --- | --- | --- | --- | --- | --- | --- |
| Alt | 1.00 | 0.20 | -0.26 | -0.48 | 0.31 | -0.07 | -0.06 |
| Alt_SD | 0.20 | 1.00 | 0.19 | 0.19 | -0.12 | -0.27 | -0.88 |
| MAP | -0.26 | 0.19 | 1.00 | 0.46 | -0.38 | 0.25 | -0.37 |
| MAT | -0.48 | 0.19 | 0.46 | 1.00 | -0.26 | 0.02 | -0.37 |
| MAT_ano | 0.31 | -0.12 | -0.38 | -0.26 | 1.00 | 0.26 | 0.13 |
| MAP_ano | -0.07 | -0.27 | 0.25 | 0.02 | 0.26 | 1.00 | 0.17 |
| Vel | -0.06 | -0.88 | -0.37 | -0.37 | 0.13 | 0.17 | 1.00 |

**Table S3. Results of** **single variable ordinary least squares linear regressions model (OLS) and simultaneously autoregressive model (SAR) of global distributions. SR: species richness; WE: weighted endemism; Vel: climate change velocity; Alt: mean altitude; Alt_SD: standard deviation of altitude; MAT: mean annual temperature; MAP: mean annual precipitation; MAT_ano: MAT anomaly; MAP_ano: MAP anomaly. Coef: Regression coefficients; R^2^ = R^2^/Nagelkerke pseudo R^2^. *p<.05, **p<.01, ***p<.001.**

|  | SR | | | | WE | | | |
| --- | --- | --- | --- | --- | --- | --- | --- | --- |
|  | OLS | | SAR | | OLS | | SAR | |
|  | Coef | R^2^ | Coef | R^2^ | Coef | R^2^ | Coef | R^2^ |
| Alt | 0.5758*** | 0.3311 | 0.1751*** | 0.8212 | 0.5641*** | 0.3178 | 0.1875*** | 0.8123 |
| Alt_SD | 0.4777*** | 0.2277 | 0.1308*** | 0.8217 | 0.5074*** | 0.257 | 0.1558*** | 0.8147 |
| MAT | 0.3332*** | 0.1105 | -0.0617 | 0.8146 | 0.4033*** | 0.1621 | -0.0187 | 0.8043 |
| MAP | 0.0882*** | 0.0071 | 0.1026** | 0.8152 | 0.1418*** | 0.0194 | 0.1347** | 0.8061 |
| MAT_ano | -0.6308*** | 0.3976 | -0.2991*** | 0.8166 | -0.6335*** | 0.401 | -0.4132*** | 0.8096 |
| MAP_ano | -0.0795** | 0.0056 | 0.0007 | 0.8142 | -0.0959*** | 0.0085 | -0.0001 | 0.8042 |
| Vel | -0.5636*** | 0.3173 | -0.1305*** | 0.8195 | -0.5826*** | 0.339 | -0.1483*** | 0.8111 |

**Table S4. Results of single variable ordinary least squares linear regressions model (OLS) and simultaneously autoregressive model (SAR) of SMT distributions. SR: species richness; WE: weighted endemism; Vel: climate change velocity; Alt: mean altitude; Alt_SD: standard deviation of altitude; MAT: mean annual temperature; MAP: mean annual precipitation; MAT_ano: MAT anomaly; MAP_ano: MAP anomaly. Coef: Regression coefficients; R^2^ = R^2^/Nagelkerke pseudo R^2^. *p<.05, **p<.01, ***p<.001.**

|  | SR | | | | WE | | | |
| --- | --- | --- | --- | --- | --- | --- | --- | --- |
|  | OLS | | SAR | | OLS | | SAR | |
|  | Coef | *R^2^* | Coef | *R^2^* | Coef | *R^2^* | Coef | *R^2^* |
| Alt | 0.613*** | 0.3736 | 0.268*** | 0.7793 | 0.4148*** | 0.1691 | 0.2677*** | 0.6591 |
| Alt_SD | 0.1988*** | 0.0361 | 0.1809*** | 0.7673 | 0.2466*** | 0.0575 | 0.2284*** | 0.6624 |
| MAT | 0.0378 | - | -0.0717 | 0.7421 | 0.0739 | 0.0019 | -0.08 | 0.623 |
| MAP | 0.0977 | 0.006 | -0.1147 | 0.7421 | 0.2233*** | 0.0464 | -0.1085 | 0.6223 |
| MAT_ano | -0.0067 | - | 0.0769** | 0.7473 | -0.058 | - | 0.0734* | 0.6266 |
| MAP_ano | 0.0664 | 0.0008 | 0.0284 | 0.7401 | 0.1464* | 0.0179 | 0.0231 | 0.6203 |
| Vel | -0.1876*** | 0.0317 | -0.1756*** | 0.7622 | -0.2344*** | 0.0516 | -0.2112*** | 0.6516 |

**Table S5. Results of single variable ordinary least squares linear regressions model (OLS) and simultaneously autoregressive model (SAR) after removing those inadequately surveyed grid cells. SR: species richness; WE: weighted endemism; Vel: climate change velocity; Alt: mean altitude; Alt_SD: standard deviation of altitude; MAT: mean annual temperature; MAP: mean annual precipitation; MAT_ano: MAT anomaly; MAP_ano: MAP anomaly. Coef: Regression coefficients; R^2^ = R^2^/Nagelkerke pseudo R^2^. *p<.05, **p<.01, ***p<.001.**

|  | SR | | | | WE | | | |
| --- | --- | --- | --- | --- | --- | --- | --- | --- |
|  | OLS | | SAR | | OLS | | SAR | |
|  | Coef | *R^2^* | Coef | *R^2^* | Coef | *R^2^* | Coef | *R^2^* |
| Alt | 0.5866*** | 0.3434 | 0.1978*** | 0.7721 | 0.5404*** | 0.2912 | 0.2442*** | 0.7276 |
| Alt_SD | 0.4824*** | 0.2318 | 0.1199*** | 0.7702 | 0.5363*** | 0.2868 | 0.1805*** | 0.7293 |
| MAT | 0.2304*** | 0.0521 | -0.092* | 0.7645 | 0.3166*** | 0.0992 | -0.0579 | 0.7148 |
| MAP | 0.032*** | - | 0.0161 | 0.7635 | 0.1206*** | 0.0134 | 0.0966 | 0.7154 |
| MAT_ano | -0.5034*** | 0.2525 | -0.1186 | 0.7642 | -0.4749*** | 0.2246 | -0.2244*** | 0.7175 |
| MAP_ano | -0.1566*** | 0.0234 | 0.0149 | 0.7635 | -0.166*** | 0.0264 | -0.0042 | 0.7144 |
| Vel | -0.514*** | 0.2633 | -0.1203*** | 0.7692 | -0.553*** | 0.305 | -0.1576*** | 0.7239 |

**Table S6.** **Partial regression for partitioning the effects of geological influences (GI), modern climate (MC) and climate stability (CS) on species richness and endemism. “GI”, “MC” and “CS”: total explained variance by “GI” or “MC” or “CS”; “GI | MC+CS”: explained variance by “GI” only; “GI+MC”: total of variance explained by both “GI” and “MC”; SR: species richness; WE: weighted endemism.**

|  | SR_Global | WE_Global | SR_SMT | WE_SMT |
| --- | --- | --- | --- | --- |
| GI | 0.38 | 0.26 | 0.27 | 0.15 |
| MC | 0.05 | 0.05 | 0.06 | 0.07 |
| CS | 0.20 | 0.16 | 0.06 | 0.07 |
| GI+MC | 0.43 | 0.29 | 0.44 | 0.29 |
| GI+CS | 0.41 | 0.28 | 0.43 | 0.27 |
| MC+CS | 0.21 | 0.17 | 0.17 | 0.14 |
| GI \| MC+CS | 0.23 | 0.14 | 0.40 | 0.22 |
| MC \| GI+CS | 0.03 | 0.03 | 0.14 | 0.09 |
| CS \| GI+MC | 0.02 | 0.01 | 0.13 | 0.07 |
| Total | 0.44 | 0.31 | 0.57 | 0.37 |
| Residuals | 0.56 | 0.69 | 0.43 | 0.63 |

**References**

Chen J, Wang YJ. 2018. New *Saussurea* (Asteraceae) species from Bogeda Mountain, eastern Tianshan, China, and inference of its evolutionary history and medical usage. PLoS One 13: e0199416.

Chen YS. 2015. Asteraceae II. *Saussurea*. – In: Hong DY *et al.* (eds), Flora of Pan-Himalaya, Vol. 48(2). Beijing: Science Press.

Martins W, Carmo W, Longo H, Couto T, Rangel T. 2013. SUNPLIN: Simulation with Uncertainty for Phylogenetic Investigations. *BMC bioinformatics,* 14: 324.

Oliveira U, Brescovit AD, Santos AJ. 2017. Sampling effort and species richness assessment: a case study on Brazilian spiders. *Biodiversity and Conservation,* 26: 1481-1493.

Raab-Straube Ev. 2017. *Taxonomic revision of Saussurea subgenus Amphilaena (Compositae, Cardueae)*. Berlin: Botanic Garden and Botanical Museum.

Rana H, Sun H, Paudel A, Ghimire SK. 2018. Saussurea ramchaudharyi (Asteraceae), a new species from Nepal. *Phytotaxa,* 340: 271-276.

Rangel TF, Colwell RK, Graves GR, Fučíková K, Rahbek C, Diniz-Filho JAF. 2015. Phylogenetic uncertainty revisited: Implications for ecological analyses. *Evolution,* 69: 1301-1312.

Sánchez-Fernández D, Lobo JM, Abellán P, Ribera I, Millán A. 2008. Bias in freshwater biodiversity sampling: the case of Iberian water beetles. *Diversity and Distributions,* 14: 754-762.

Shi Z, Raab-Straube EV. 2011. *Flora of China* (Asteraceae). 20–21: 1–992. In Wu C Y *et al.* eds. *Flora of China*. Beijing & St. Louis: Science Press & Missouri Botanical Garden Press.

Shrestha N, Wang Z, Su X*, et al.* 2018. Global patterns of Rhododendron diversity: The role of evolutionary time and diversification rates. *Global Ecology and Biogeography,* 27: 913-924.

Xu L-S, Herrando-Moraira S, Susanna A, Galbany-Casals M, Chen Y-S. 2019. Phylogeny, origin and dispersal of *Saussurea* (Asteraceae) based on chloroplast genome data. *Molecular Phylogenetics and Evolution,* 141: 106613.

Xu X, Dimitrov D, Shrestha N, Rahbek C, Wang Z, Jordan G. 2019. A consistent species richness–climate relationship for oaks across the Northern Hemisphere. *Global Ecology and Biogeography*: 28: 1-16.

Zhang Y, Tang R, Huang X, Sun W, Ma X, Sun H. 2019. *Saussurea balangshanensis* sp. nov. (Asteraceae), from the Hengduan Mountains region, SW China. *Nordic Journal of Botany,* 37: 1-7.
